# Prevalence of MTP joint involvement in the painful forefoot of patients with Rheumatoid Arthritis

**DOI:** 10.1186/1757-1146-3-S1-O23

**Published:** 2010-12-20

**Authors:** Heidi Siddle, Anthony Redmond, Richard Wakefield, Richard Hodgson, Andrew Grainger, David Pickles, Philip Helliwell

**Affiliations:** 1 University of Leeds, Leeds, UK; 2 Leeds Musculoskeletal Biomedical Research Unit, Leeds, UK; 3 Leeds Teaching Hospitals NHS Trust, Leeds, UK

## Introduction

Despite the introduction of more aggressive systemic therapies for the management of rheumatoid arthritis (RA) MTP joint pain & deformity is commonly reported. Previous research has proposed that articular damage in the forefoot of patients with RA is probably caused by synovitis leading to destruction of the bone & soft tissues, which might result in the failure of a complex ligamentous system & the dynamic effect of displacement of the plantar plates (PP).

## Methods

In 24 patients with RA the more symptomatic forefoot was imaged using MRI, high-resolution ultrasound (HRUS) & conventional radiography (CR). The MRI & HRUS images were assessed to determine inflammation & damage at the MTP joints, the presence or absence of the PP & to identify tears of the PP & their location. CR was used to determine radiographic progression at the MTP joints.

## Results

17 females and 7 males (mean (SD) age of 55.5 (10.5) years, disease duration 10.6 (8.6) years & forefoot pain score was 43.4 (27.9)) took part.

Tears were not identified on HRUS. The Larsen score on the index foot correlated with the absence of PP viewed using both MRI (r=0.499,p=0.013) & HRUS (r=0.751,p<0.001). MRI synovitis was not related to PP absence on either MRI (r=0.187,p=0.381) or HRUS (r=0.154,p=0.472) but there was good correlation between MRI bone oedema & the number of absent PP visualised using MRI (r=0.525,p=0.028) & HRUS (r=0.729,p<0.001). There was also a strong correlation between MRI imaged bone erosion & PP absence confirmed using both MRI (r=0.613,p=0.001) & HRUS (r=0.737,p<0.001).

## Discussion

In agreement with existing literature, this current study demonstrates that pain & deformity at the MTP joints is highly prevalent despite good disease control. The findings indicate that PP failure is associated more with damage at the lesser MTP joints than active synovitis in patients with RA. Longitudinal follow-up of patients with RA & forefoot pain is required to establish the relationship between local inflammation, disease activity, impairment & structural changes, & identify factors that precede & result in PP pathology.

